# An online database for plant image analysis software tools

**DOI:** 10.1186/1746-4811-9-38

**Published:** 2013-10-09

**Authors:** Guillaume Lobet, Xavier Draye, Claire Périlleux

**Affiliations:** 1 Laboratory of Plant Physiology, PhytoSYSTEMS, University of Liège, Boulevard du Rectorat 27, 4000 Liège, Belgium; 2 Earth and Life Institute, Université catholique de Louvain, Croix du Sud 2 L7.05.11, 1348, Louvain-la-Neuve, Belgium

**Keywords:** Database, Image analysis, Social media

## Abstract

**Background:**

Recent years have seen an increase in methods for plant phenotyping using image analyses. These methods require new software solutions for data extraction and treatment. These solutions are instrumental in supporting various research pipelines, ranging from the localisation of cellular compounds to the quantification of tree canopies. However, due to the variety of existing tools and the lack of central repository, it is challenging for researchers to identify the software that is best suited for their research.

**Results:**

We present an online, manually curated, database referencing more than 90 plant image analysis software solutions. The website, plant-image-analysis.org, presents each software in a uniform and concise manner enabling users to identify the available solutions for their experimental needs. The website also enables user feedback, evaluations and new software submissions.

**Conclusions:**

The plant-image-analysis.org database provides an overview of existing plant image analysis software. The aim of such a toolbox is to help users to find solutions, and to provide developers a way to exchange and communicate about their work.

## Background

Many fields of plant sciences, ranging from physiological studies to breeding programs, rely on linking genotypes and phenotypes. Thanks to the increasing development of genotyping techniques, plant scientists have been generating an incredibly large amount of genetic data. However, to effectively use this genetic information, it must be explicitly linked, together with environmental characterisation, to the corresponding phenotypic responses. Unfortunately, the lack of appropriate phenotyping tools and methods often hinders these studies, making the phenotyping step the bottleneck in many research programs [1].

Current phenotyping pipelines often rely on imaging techniques [2,3]. Indeed, images have several key features that make them valuable for plant research: they are multidimensional in nature, contain several layers of information (e.g. shape and colors), allow a temporal decoupling of sampling and analysis and are prone to standardized and fully automated treatment. In addition, with the widespread adoption of simple imaging tools [4,5], with the appearance of more complex technologies [6-8] and with the increasing availability of powerful hardware, a majority of plant scientists use one or several imaging techniques in their research.

Computer scientists and plant biologists have been developing software solutions to handle imaging data [9]. Most of the time, these developments were bound to specific applications, for example the analysis of shoot meristem functioning [10] or the 3D reconstruction of entire root systems [11]. Unfortunately, the ever increasing number of available tools and the diversity of communication means within the scientific community, make it difficult for the non-specialists to find the most appropriate solution for their analyses. We therefore created a new online database referencing available plant image analysis software and allowing new tools deposit. This paper describes the database which aims to bridge the gap between software developers and users and help scientists to find the tools they need.

## Construction and content

### General structure

**Figure 1:**
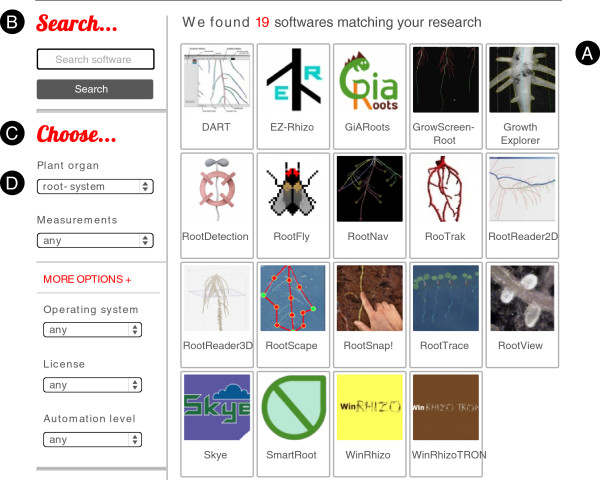
**Main search page of the plant-image-analysis.org website.** Users can browse through the software solutions **(A)**, make a free search **(B)**, or use pre-defined search criteria **(C)**. Here, the list of software was restricted by the application of a filter on the organ type *root-system***(D)**.

The plant image analysis database was built as a web-based repository and is freely available at the following address: www.plant-image-analysis.org [12]. The web interface was designed to enable scientists to quickly identify the right tools for their research (Figure 1). Users can navigate through the complete list of software solutions, query the database with keywords or browse it with pre-defined criteria such as the type of organ to analyse, the measurements to make, the desired automation level, the operating system or the license type. Users can stay informed using either RSS fluxes or following the database Twitter account (@plant_image).

### Data sources and management

**Figure 2:**
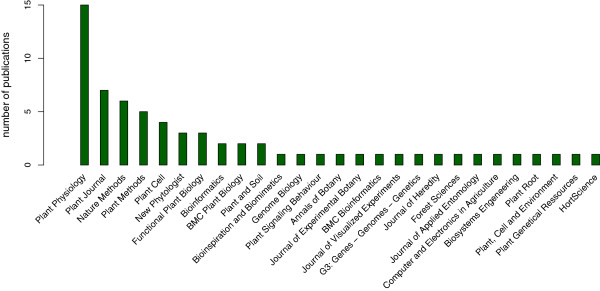
**Number of publications presenting plant image analysis software per journal.** Total number of journals = 27.

Because the information about existing plant image analysis software is usually scattered among a variety of scientific publications (Figure 2) and websites, it is virtually impossible to automate data collection and database feeding. A manual curation of the database is therefore required to ensure that most of the available tools are presented and properly classified. The presence of broken links is periodically checked using Google Webmaster tools [13].

Both published and unpublished software were found through a thorough review of the literature and by using scientific reference databases (such as Scopus [14], ISI Web of Knowledge [15] or Google Scholar [16]) and regular search engines (such as Google). Although the list may not be exhaustive, we believe that most of the major plant image analysis software were found and incorporated. Up-dating the database is performed by automated web queries, literature screening (with a focus on journals where plant imaging tools were published, Figure 2) and developer contributions. Any software can be added on request by sending a predefined form to the database curators.

Regarding the database management, each software was assigned a set of keywords describing the target organ, measured parameters, automation level, license type and operating system. In order to avoid duplication and ambiguity, a limited number of keywords were selected. These keywords were chosen to describe the most basic features extracted from the images, excluding their combinations. As an example, for rosette analysis in Arabidopsis, “compactness” (defined as the ratio between rosette area and convex hull area, [17,18]) was not retained since it could be easily recalculated from its basic components. However, the number of keywords is not fixed and new features can be added if needed.

### Software presentation and description

**Figure 3:**
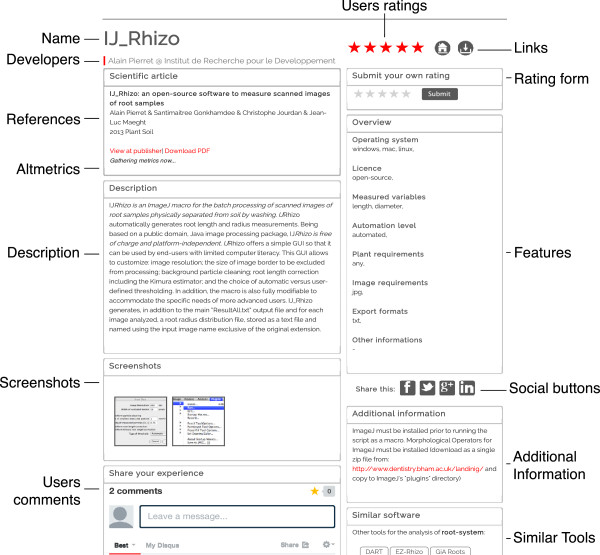
**Software presentation page on the plant-image-analysis.org website.** Software presentation page on the plant-image-analysis.org website (in this case, IJ-Rhizo’s presentation page [19]).

The plant-image-analysis.org database is organized as a set of presentation sheets describing the different software in a concise and homogenized style (Figure 3). Firstly, a short description introduces each software, generally based on the information provided by its developer. Secondly, a formatted list of the main software features is reported including the plant part for which it was designed, the nature of the collected data, the level of automation, the operating system, the license type, the plant and image requirements and the export format. This set of information was chosen to meet the criteria usually used by researchers when searching for a software solution. References of related publications are given (if any), as well as the name of the developers.

In addition, taking advantage of recent web technologies, user feedback and software evaluations have been implemented in every presentation page. This layer of information, fed by the scientists themselves, aims at improving the tools and establishing interactions between software users and developers. Social media links (such as Twitter or Facebook) have been added to promote sharing and discussions [20]. Finally, altmetrics (alternative **metrics**[21]) have been added for every published article using a widget developed by ImpactStory [22]. These metrics convey information on the articles reception by the scientific community and provide the users with an indication of the relevancy of the paper in their field [23].

## Utility and discussion

Currently, the database references more than 90 software, ranging from cell to whole canopy analyses. It provides a way for developers of plant image analysis tools to present their work, published or unpublished. It enables plant scientists to easily find and compare the different tools available for their research. Since its release in January 2013, the website received an average of 500 visitors per month (data retrieved from Google Analytics [24]), confirming the interest of the scientific community for such a repository.

### An overview of the available plant image analysis software

**Figure 4:**
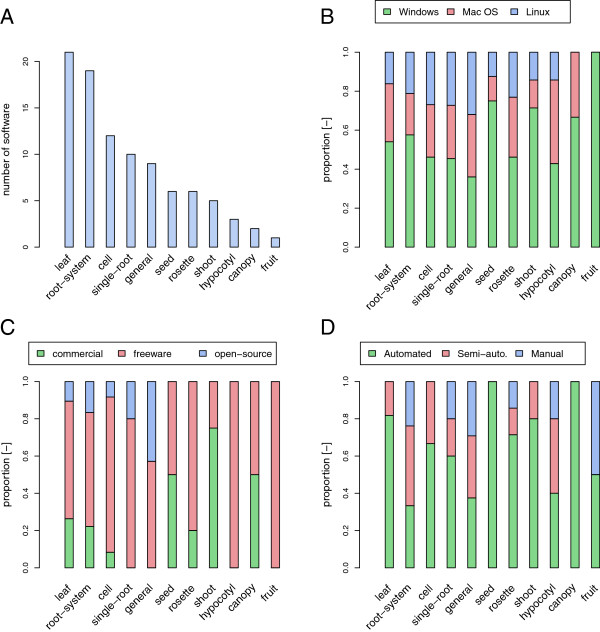
**Distribution of the tools presented in the plant-image-analysis.org website.** Distribution of the tools presented in the plant-image-analysis.org website, by plant organ type. **A**. Number of software by plant organ type. **B**. Proportion of operating systems by organ type. **C**. Proportion of license type by organ type. **D**. Proportion of automation levels by organ type. Total number of software = 91.

The plant-image-analysis.org database allows to draw an overview of the available plant image analysis tools (Figure 4).

A first general observation is that the number of available systems varies very much with plant organs (Figure 4A). In particular, a large proportion of the tools are dedicated to individual leaves, then to the analysis of roots (either root systems or single roots) and cells. As stated earlier, the important number of similar, yet different, tools reflects the diversity of biological questions and hardware solutions. Software solutions are frequently developed in a specific context, making them unsuitable for other applications. Fortunately, developers have begun to address this issue as demonstrated by the recent publication of more flexible software solutions. As an example, for the analysis of rosette of Arabidopsis plants, Rosette Tracker provides a framework adaptable to multiple experimental designs [17]. Still for the analysis of rosette (or individual leaves), PhenoPhyte is accessible through a web-interface, making it a easy cross-platform solution that does not require any installation on a local machine [25]. For the analysis of root system, both SmartRoot [26] and RootNav [27] rely on semi-automated root tracing procedures that make them suitable for a large range of image types and qualities thanks to their semi-automated root tracing process.

While many plant image analysis applications were originally developed for the Windows operating system (Figure 4B), it should be noted that many developers have made efforts to offer cross-platform solutions. Moreover, a large majority of the referenced software are available for free for the scientific community (and even open-source, Figure 4C). Both the inter-operability between operating systems and the free access highlight positive dynamics in the on-going development of new plant image analysis software.

Finally, in line with the growing development of phenotyping platforms and pipelines, a clear tendency towards a full automation of the image analyses process can be observed (Figure 4D). Interestingly, for few organ types such as the root systems, a fair proportion of manual or semi-automated tools are available. In the case of the analysis of root systems, this distribution can be explained, at least partially, by the intrinsic nature of the objects to analyse. Root systems are indeed highly branched and complex structures, which makes automated analysis challenging and prone to cumulative errors [27]. In such scenario, semi-automated and manual procedures are still needed for the acquisition of quality data.

**Figure 5:**
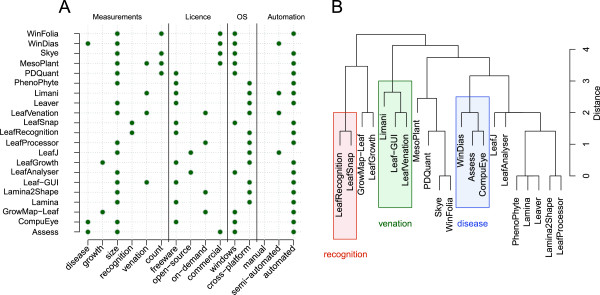
**Illustration of the redundancy between the existing plant image analysis software solutions.** Illustration of the redundancy between the existing plant image analysis software solutions, here for the analysis of single leaves. **A**. Properties of each software (measurements, license, operating system and automation level). **B**. Clustering of the different tools, based on their properties. A higher weight was given to the measurements compared to the other properties for the clustering (2:1). Tools designed for specific measurements (disease, venation analysis or species recognition) are highlighted in colors. For both figures, and for the sake of clarity, measurements were pooled by classes (e.g. *size* encompasses surface, length, width and perimeter measurements).

Our analysis shows that, in many cases, new users will have the choice between different software solutions (Figure 5A) that were developed independently for specific purposes and with given constrains. Although these tools present some redundancy (Figure 5B), they also contain complementarity approaches that could be combined for further improvement. By providing a classified and homogenised presentation of the available image analysis software solutions, the plant-image-analysis.org database aims at increasing the communication between developers, so participating to the concerted development of future tools.

## Conclusion

The variety of biological questions, hardware solutions and technical approaches in plant image analysis have led to the development of a wide variety of tools and software. The diversity of hosting solution (from personal webpages to centralized repositories) and publication type (from none to biological to computational journals) has led to the dispersion of these tools across the web, making it difficult for a researcher to find the right tool for his research.

Here we presented a new online, manually curated, database that references and presents more than 90 plant image analysis tools. This database enables developers to present their tools (both published or unpublished) and users to easily navigate through the space of possible software solutions to find the most appropriate solution for their research.

## Availability and requirements

The plant image analysis software database is freely available at the address: http://www.plant-image-analysis.org and is compatible with all major web browsers.

## Competing interests

The authors declare that they have no competing interests.

## Authors’ contributions

GL designed, filled and still maintains the database. XD and CP supervised the project. GL, XD and CP wrote the manuscript. All authors read and approved the final manuscript.
